# Effect of high-dose vitamin C therapy on severe burn patients: a nationwide cohort study

**DOI:** 10.1186/s13054-019-2693-1

**Published:** 2019-12-12

**Authors:** Mikio Nakajima, Morita Kojiro, Shotaro Aso, Hiroki Matsui, Kiyohide Fushimi, Yasuhiko Kaita, Hideaki Goto, Yoshihiro Yamaguchi, Hideo Yasunaga

**Affiliations:** 1 Emergency and Critical Care Center, Tokyo Metropolitan Hiroo Hospital, 2−34−10, Ebisu, Shibuya-ku, Tokyo, 150-0013 Japan; 2 Department of Clinical Epidemiology and Health Economics, School of Public Health, The University of Tokyo, Tokyo, Japan; 3 Department of Trauma and Critical Care Medicine, Kyorin University School of Medicine, Tokyo, Japan; 4 Department of Health Policy and Informatics, Tokyo Medical and Dental University Graduate School of Medicine, Tokyo, Japan

**Keywords:** Burn, Vitamin C, Ascorbic acid, Propensity score, Matching, Competing risk

## Abstract

**Background:**

Vitamin C is a well-documented antioxidant that reduces oxidative stress and fluid infusion in high doses; however, the association between high-dose vitamin C and reduced mortality remains unclear. This study evaluates the effect of high-dose vitamin C in severe burn patients under two varying thresholds.

**Methods:**

We enrolled adult patients with severe burns (burn index ≥ 15) who were registered in the Japanese Diagnosis Procedure Combination national inpatient database from 2010 to 2016. Propensity score matching was performed between patients who received high-dose vitamin C within 1 day of admission (vitamin C group) and those who did not (control group). High-dose vitamin C was defined as a dosage in excess of 10 g or 24 g within 2 days of admission. The primary outcome was in-hospital mortality.

**Results:**

Eligible patients (*n* = 2713) were categorized into the vitamin C group (*n* = 157) or control group (*n* = 2556). After 1:4 propensity score matching, we compared 157 and 628 patients who were administered high-dose vitamin C (> 10-g threshold) and controls, respectively. Under this particular threshold, high-dose vitamin C therapy was associated with reduced in-hospital mortality (risk ratio, 0.79; 95% confidence interval, 0.66–0.95; *p* = 0.006). In contrast, in-hospital mortality did not differ between the control and high-dose vitamin C group under the > 24-g threshold (risk ratio, 0.83; 95% confidence interval, 0.68–1.02; *p* = 0.068).

**Conclusions:**

High-dose vitamin C therapy was associated with reduced mortality in patients with severe burns when used under a minimum threshold of 10 g within the first 2 days of admission. While “high-dose” vitamin C therapy lacks a universal definition, the present study reveals that different “high-dose” regimens may yield improved outcomes.

## Introduction

Severe burns require appropriate fluid management in the acute phase [1]. A massive volume of intravenous fluid is usually required to ensure adequate end-organ perfusion. However, increased capillary permeability allows escape of intravascular fluid and proteins into the interstitial space. Reactive oxygen species contribute to increased endothelial permeability [2].

Vitamin C, or ascorbic acid, is an inexpensive and readily available antioxidant commonly deployed in the clinical settings [1]. Experimental studies showed that vitamin C can decrease oxidative stress in endothelial cells and tighten endothelial barriers [2, 3]. Several preclinical studies and two clinical studies demonstrated that high-dose vitamin C can reduce fluid infusion and subsequent edema [4–8]. However, the two clinical studies had relatively small numbers of patients (*n* = 37 and *n* = 33) and their measured outcome was only fluid saving in the first 24 h [7, 8]. To our knowledge, no studies have demonstrated an association between high-dose vitamin C and reduced mortality. Furthermore, a question that remains unanswered in this context is the optimum dosage of “high-dose” vitamin C therapy in burn patients as there is no universally adopted definition [9].

The present study aimed to evaluate the effect of high-dose vitamin C in patients with severe burns under two different thresholds of “high-dose” vitamin C, using a nationwide inpatient database in Japan.

## Methods

### Data source

A nationwide cohort study was carried out using the Diagnosis Procedure Combination database described in previous reports [10–12]. The database in question encompasses a nationwide spectrum, includes administrative claims data and discharge abstracts from more than 1200 acute-care hospitals, and covers approximately 90% of all tertiary-care emergency hospitals in Japan. The database also includes the following data for each patient: admission/discharge dates, age, sex, body weight at admission, level of consciousness and comorbidities on admission, diagnoses, and complications during hospitalization, recorded under the International Classification of Diseases, Tenth Revision (ICD-10) codes and text data entered in Japanese. Medical procedures, daily records for drugs, blood products/devices used, and discharge status were also included. Consciousness level on admission was evaluated via the Japan Coma Scale (JCS) score defined as follows: 0, alert consciousness; 1–3, awake without any stimuli; 10–30, aroused by some stimuli; and 100–300, coma. Assessments by the JCS and Glasgow Coma Scale have been evaluated in the past and the general correlation thereof confirmed via multiple studies [13, 14]. In lieu of total burn surface area (TBSA), a burn index that reflects both the surface area and the thickness of the burn is also included in the database and can be calculated as follows [11]: burn index = full thickness of TBSA + 1/2 partial thickness of TBSA. Large studies conducted to date have collectively suggested that the burn index was a good predictor of mortality in burn patients [11, 15]. The ICD-10 code for each comorbidity was converted to a score to quantify the extent of the comorbidities, and the sum of these scores was used to calculate the Charlson comorbidity index (CCI) [16]. The CCI has seen widespread use to measure case mixes and disease burdens [17]. CCI was categorized into three groups as previously reported [11]: low, 0; medium, 1; and high, ≥2.

### Patient selection

We extracted data for patients discharged from the hospital between July 2010 and March 2016 with a primary diagnosis of burns (ICD-10 codes: T20–T32). Patients aged ≥ 15 years with burn index ≥ 15 were included [18]. In contrast, patients who were discharged within 1 day after admission (to avoid immortal time bias) were excluded. Additionally, patients with first admission were included whereas readmitted patients were excluded from the study.

We compared patients who received high-dose vitamin C within 1 day after admission (high-dose vitamin C group) and those who did not receive high-dose vitamin C (control group). In this study, high-dose vitamin C was administered under two varying thresholds: dosage in excess of (1) 10 g within 2 days of admission [9, 19] and (2) 24 g within 2 days of admission [20, 21]. In general, high-dose vitamin C therapy is only administered as a continuous intravenous infusion during the initial 24-h period after admission [7].

### Outcomes

The primary outcome was all-cause in-hospital mortality. The secondary outcomes included total fluid volume within 1, 3, and 7 days of admission.

### Statistical analysis

Continuous variables were reported as median and interquartile range (IQR), and categorical variables were reported as number and percentage.

To account for differences in baseline characteristics between patients with and without high-dose vitamin C, we performed a propensity score analysis. A logistic regression model was performed to calculate propensity scores for patients receiving high-dose vitamin C, using the following patient background characteristics and interventions performed within 1 day of admission [11, 15, 22–25]: age, sex, burn index, presence of inhalation injury, CCI, JCS, use of vasopressor, albumin, hydroxyethyl starch, intravenous antibiotics, neuromuscular blockade, haptoglobin, transfusion (red blood cells, platelets, and fresh frozen plasma), mechanical ventilation, renal replacement therapy (RRT), implementation of pharyngolaryngeal/bronchial/gastrointestinal endoscopy, enteral feeding via nasogastric tube, inta-arterial blood pressure monitoring, utilization of intensive care unit, admission to teaching hospital, and transportation from another hospital. A one-to-four propensity score matching was next performed by nearest-neighbor matching with replacement. The width of the caliper was set at 20% of the standard deviation of the propensity scores on the logit scale. Balances in baseline variables using standardized differences were also examined. Absolute values of < 10% were considered balanced [26].

For in-hospital mortality, risk ratios for the high-dose vitamin C group compared with the control group were calculated in the propensity-matched group. Total fluid volumes within 1, 3, and 7 days of admission were compared between the groups using Wilcoxon’s rank-sum test in the matched cohort. Two-sided *p* values of < 0.05 were considered significant. All analyses were performed using Stata MP15 (STATA Corp, College Station, TX).

## Results

### High-dose defined as > 10 g of vitamin C

**Fig. 1:**
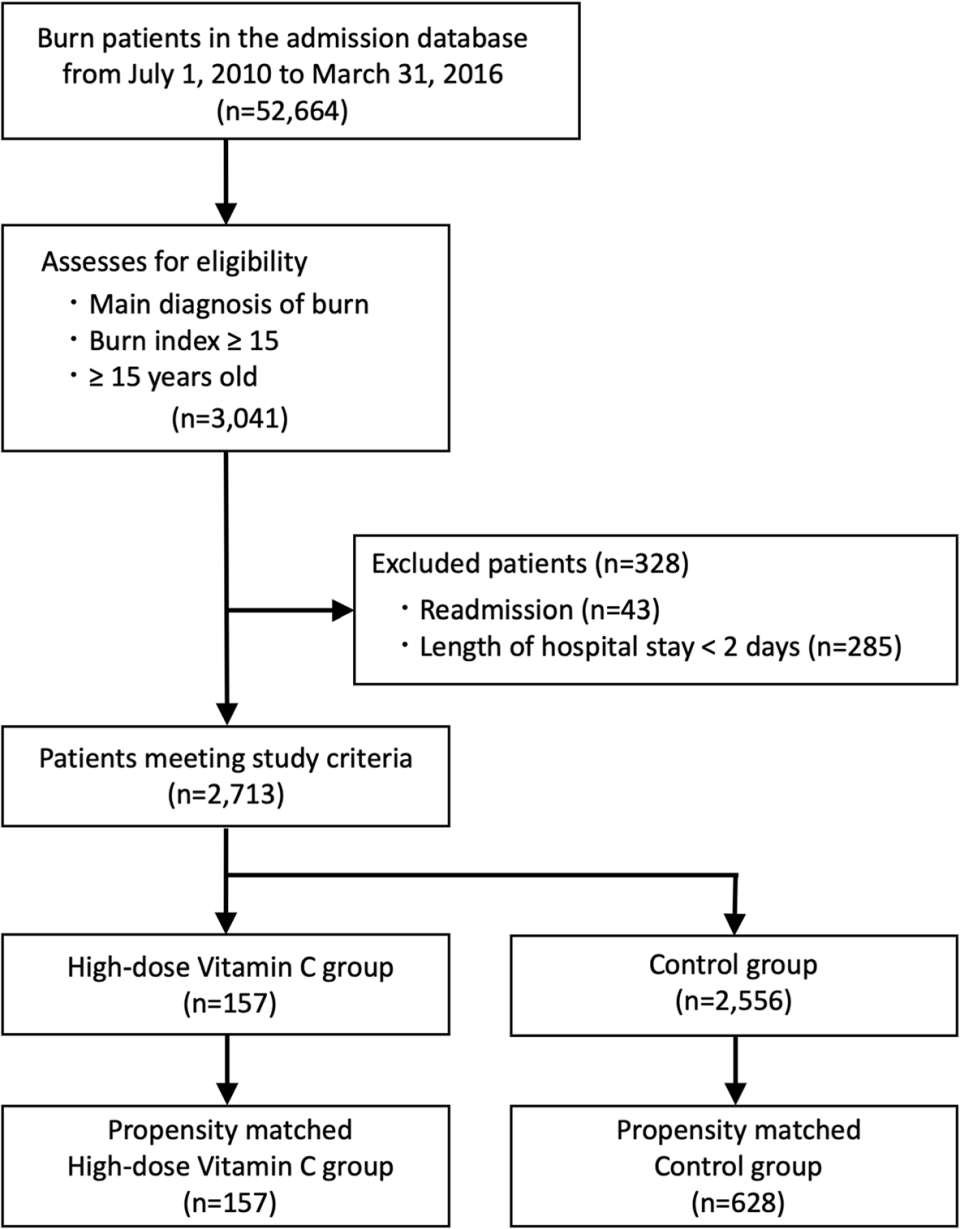
Patient selection (10 g minimum threshold of high-dose vitamin C)

We identified 2713 patients after application of the inclusion and exclusion criteria (Fig. 1). The patients were divided into the high-dose vitamin C group (*n* = 157) and control group (*n* = 2556). After 1:4 propensity score matching, we compared 157 and 628 patients who were administered high-dose vitamin C and controls, respectively. The C-statistic was 0.79.

Table 1 shows the baseline characteristics of the unmatched and propensity score-matched groups. Patients were more likely to receive vitamin C if they were of younger age; had higher body weight and burn index; were in a comatose state; presented with inhalation injuries; were administered vasopressors, albumin, hydroxyethyl starch, neuromuscular blockade, haptoglobin, or transfusion; underwent surgery or endoscopy; or were placed under mechanical ventilation, renal replacement therapy, or intra-arterial blood pressure monitoring within 1 day after admission. The vitamin C group displayed a tendency of being admitted to a teaching hospital and intensive care units. The patient characteristics were well-balanced between the two groups after propensity score matching. The median dose of vitamin C administered was 50 g (IQR, 26–83 g) in the vitamin C group and 0 g (IQR, 0–0 g) in the control group within a 2-day period. There was no missing data for the variables adopted in this study.

**Table 1:** Baseline patient characteristics before and after propensity score matching (10 g minimum threshold of high-dose vitamin C)

Variables	Before propensity score matching			After propensity score matching		
	High-dose vitamin C group (*n* = 157)	Control group (*n* = 2556)	Standardized difference (%)	High-dose vitamin C group (*n* = 157)	Control group (*n* = 628)	Standardized difference (%)
Age(years), median (IQR)	67 (48–80)	69 (51–80)	11.7	67 (48–80)	67.5 (50–80)	7.6
Male, *n* (%)	87 (55.4)	1561 (61.1)	11.5	87 (55.4)	332 (52.9)	5.1
Body weight (Kg), median (IQR)	58.5 (47.4–68.9)	55.4 (45–65)	23.4	58.5 (47.4–68.9)	57.15 (50–69)	2.6
Burn index, median (IQR)	34 (24–50)	25 (18–40)	39.3	34 (24–50)	34.5 (23–53.4)	1.1
Inhalation injury, *n* (%)	45 (28.7)	523 (20.5)	19.1	45 (28.7)	181 (28.8)	0.4
Charlson comorbidity Index, *n* (%)						
0 (low)	126 (80.3)	1917 (75.0)	12.6	126 (80.3)	484 (77.1)	7.8
1 (medium)	24 (15.3)	444 (17.4)	5.6	24 (15.3)	115 (18.3)	8.1
≥ 2 (high)	7 (4.5)	195 (7.6)	13.3	7 (4.59)	29 (4.6)	0.8
Japan Coma Scale, *n* (%)						
0 (alert)	69 (43.9)	1406 (55.0)	22.3	69 (43.9)	266 (42.4)	3.2
1–30 (dizziness)	38 (24.2)	553 (21.6)	6.1	38 (24.2)	145 (23.1)	2.6
10–30 (somnolence)	6 (3.8)	173 (6.8)	13.2	6 (3.8)	28 (4.5)	3.2
100–300 (coma)	44 (28.0)	424 (16.6)	27.7	44 (28.0)	189 (30.1)	4.6
Prescriptions within 1 day, *n* (%)						
Vasopressor use	44 (28.0)	441 (17.3)	25.9	44 (28.0)	180 (28.7)	1.4
Albumin use	74 (47.1)	530 (20.7)	57.9	74 (47.1)	285 (45.4)	3.5
Hydroxyethyl starch use	13 (8.3)	122 (4.8)	14.2	13 (8.3)	43 (6.8)	5.4
Intravenous antibiotics use	70 (44.6)	1159 (45.3)	1.5	70 (44.6)	289 (46.0)	2.9
Neuromuscular blockade use	63 (40.1)	565 (22.1)	39.6	63 (40.1)	254 (40.4)	0.6
Haptoglobin use	59 (37.6)	378 (14.8)	53.6	59 (37.6)	242 (38.5)	2.0
Transfusion use	34 (21.7)	212 (8.3)	38.0	34 (21.7)	132 (21.0)	1.6
Procedures within 1 day, *n* (%)						
Operation	61 (38.9)	438 (17.1)	49.7	61 (38.9)	243 (38.7)	0.3
Mechanical ventilation	114 (72.6)	1180 (46.2)	55.8	114 (72.6)	474 (75.5)	6.5
Renal replacement therapy	8 (5.1)	71 (2.8)	11.9	8 (5.1)	27 (4.3)	3.8
Endoscopy	54 (34.4)	448 (17.5)	39.1	54 (34.4)	212 (33.8)	1.3
Enteral feeding	11 (7.0)	202 (7.9)	3.4	11 (7.0)	47 (7.5)	1.8
Intra-arterial blood pressure monitoring	123 (78.3)	1161 (45.4)	71.9	123 (78.3)	492 (78.3)	0.1
Admission site, *n* (%)						
Teaching hospital	156 (99.4)	2436 (95.3)	25.4	156 (99.4)	623 (99.2)	1.9
Intensive care unit	155 (98.7)	2059 (80.6)	62.4	155 (98.7)	622 (99.0)	3.0
Transportation from another hospital, *n* (%)	7 (4.5)	124 (4.9)	1.9	7 (4.5)	35 (5.6)	5.1

*IQR* interquartile range

### High-dose defined as > 24 g of vitamin C

We identified 2713 patients during the target period after application of the inclusion and exclusion criteria (Additional file 1: Fig. S1). The patients were divided into the high-dose vitamin C group (*n* = 127) and control group (*n* = 2586). After 1:4 propensity score matching, we compared 127 and 508 patients who were and were not administered high-dose vitamin C, respectively. The C-statistic was 0.81.

Similar to the analysis using the 10-g threshold above, patients in more serious conditions had an increased likelihood to receive vitamin C (Additional file 2: Table S1). The patient characteristics were similar between the two groups after propensity score matching. The median dose of vitamin C administered was 63 g (IQR, 39–92 g) in the vitamin C group and 0 g (IQR, 0–0 g) within a 2-day period.

### In-hospital mortality

**Fig. 2:**
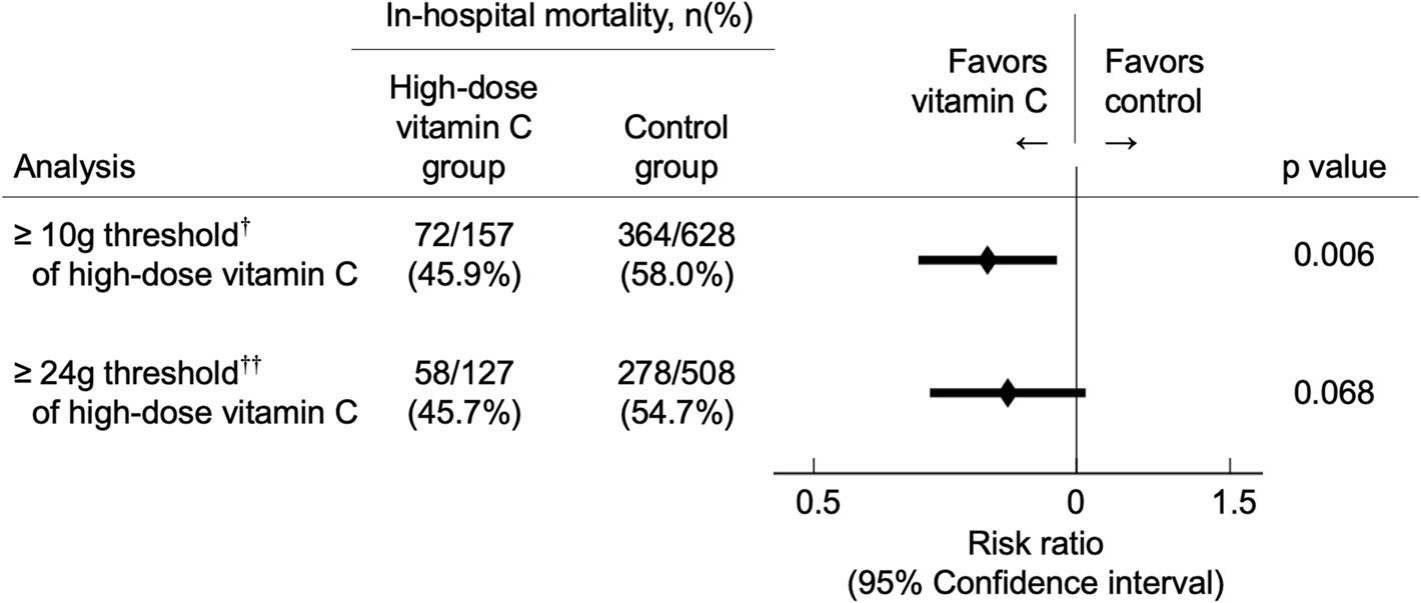
Primary outcomes under varying high-dose vitamin C thresholds after propensity score matching, ^†^10-g minimum threshold within 2 days of admission, ^††^24-g minimum threshold within 2 days of admission

The results of the primary outcome after propensity score matching are shown in Fig. 2. In-hospital mortality was significantly reduced when applying the 10-g threshold (risk ratio, 0.79; 95% confidence interval, 0.66–0.95). In contrast, in-hospital mortality did not differ significantly between the two groups when applying the 24-g threshold.

### Total fluid volume

**Fig. 3:**
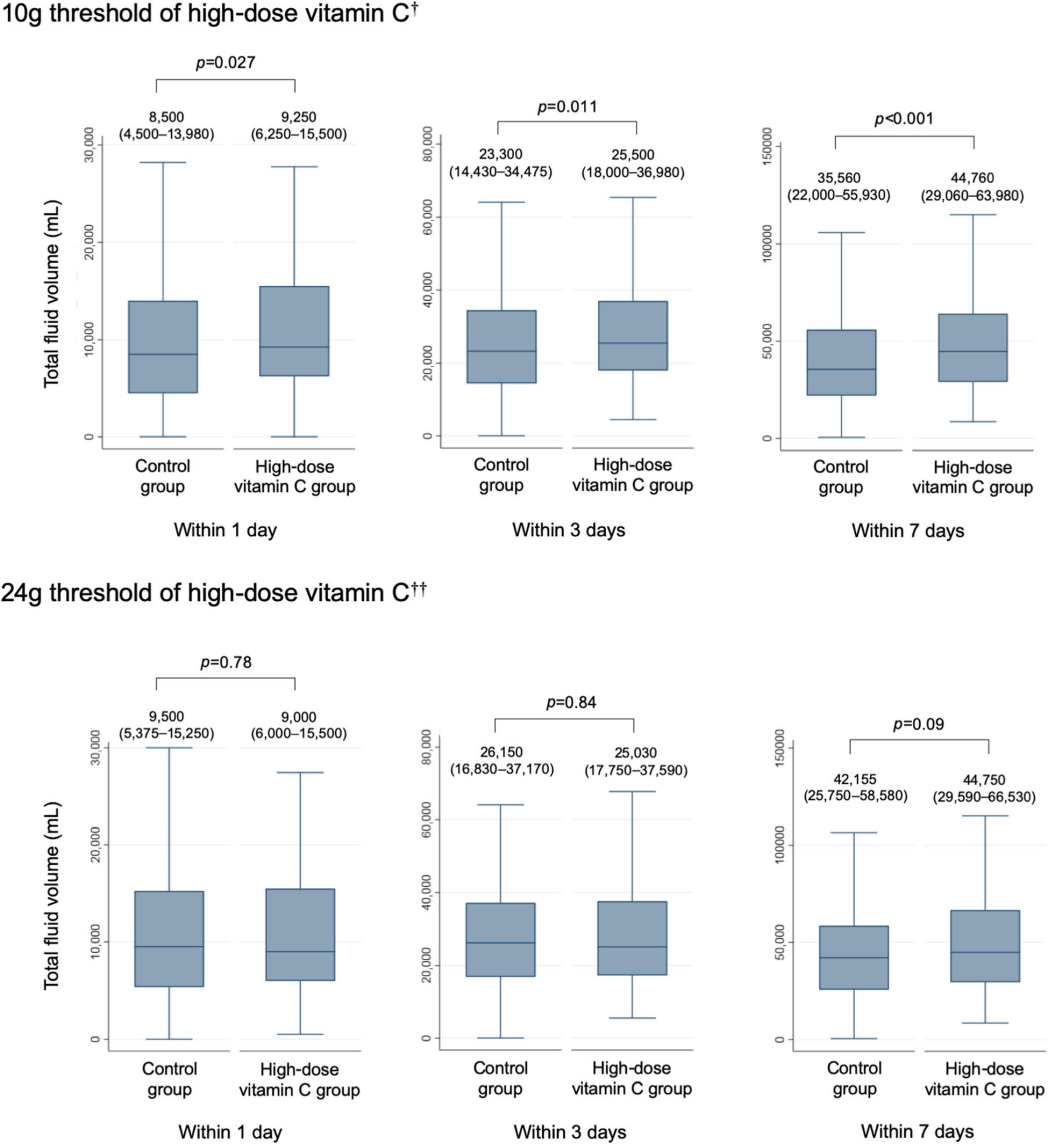
Secondary outcomes after propensity-score matching under varying high-dose vitamin C thresholds, ^†^10-g minimum threshold within 2 days of admission, ^††^24-g minimum threshold within 2 days of admission

The results for the secondary outcomes after propensity score matching are shown in Fig. 3. The high-dose vitamin C group showed a significantly higher total fluid volume within 1, 3, and 7 days compared with the control group under the 10 g minimum threshold of vitamin C. Under the 24 g minimum threshold, total fluid volume within 1, 3, and 7 days were similar between the two groups.

## Discussion

In the present study, high-dose vitamin C therapy was significantly associated with reduced in-hospital mortality in patients administered a 10-g minimum threshold of high-dose vitamin C compared with those who were not administered high-dose vitamin C. On the contrary, under the 24-g threshold of high-dose vitamin C, in-hospital mortality did not differ between the groups.

Although several preclinical studies demonstrated the potential benefit of high-dose vitamin C [2, 4–6], only two clinical studies have examined the utility of high-dose vitamin C as an adjunct in burn resuscitation. Both studies were limited by small sample sizes and residual confounding biases. A randomized controlled trial in 2000 showed that the vitamin C group required less fluid during the first 24-h period and shorter duration of mechanical ventilation compared with the non-vitamin C group [7]. However, they did not find a significant difference in mortality. The weaknesses of the study were the small sample size (*n* = 37) and high risk of type-2 errors. Furthermore, the patients in the vitamin C group were younger and had smaller TBSA and fewer fasciotomies than the control group. Meanwhile, a retrospective observational study found a reduced 24-h fluid volume in the vitamin C group compared with the control group but did not show differences in respiratory function or mortality [8]. That study also had weaknesses, including small sample size (*n* = 33) as well as younger age and smaller TBSA in the vitamin C group compared with the control group. The present study is the first to reveal significant differences in mortality after carefully adjusting for numerous confounding factors using propensity score matching varying vitamin C threshold.

Tanaka et al. first defined high-dose vitamin C as 66 mg/kg/h in severe burn patients [7]. This very high dosage was based on animal experiments and it remains unknown if such a high dose is clinically necessary. We therefore applied the definition for high-dose vitamin C used in a real-world clinical setting (1 g/h = 24 g/day). However, an important counterargument to consider is that the vitamin C dosage we adopted in the present study may have been too small to exert any tangible effects. Outside the spectrum of burn patients, numerous studies have adopted varying dosages of vitamin C as “high-dose” in critically ill patients [9, 19]. Therefore, there is no universal definition of “high-dose” vitamin C and thus we adopted two varying minimum thresholds of high-dose vitamin C in this study.

Although the 10-g threshold of high-dose vitamin C demonstrated a significant reduction in mortality, the point estimate under the ≥ 24-g threshold was 0.83 and 95% CI was 0.68–1.02. Ergo the reason in which no statistical significance is achieved is likely dependent on the number of participants included in the study (type-2 error). Therefore, the argument can be made that if more patients were included in the study, statistical significance could perhaps have been achieved.

Previous studies have demonstrated the fluid saving effect of high-dose vitamin C as a primary outcome as defined as total fluid volume within 24 h [7, 8]. Contrary to the previous studies, our study showed larger or similar total fluid volume within 1, 3, and 7 days of admission. It is important to note that total fluid volume in observational studies can contain competing risks. Patients who expired within a relatively short timeframe after admission appear to have necessitated a smaller amount of total fluid volume; however, in reality, the patient received a lower fluid volume due to early expiration—in these cases, death becomes an important competing risk to consider.

There are several limitations associated with this study that should be noted. The present study was a non-randomized observational study and hence suffered from potential selection and ascertainment biases, despite careful mitigation via propensity score matching. First, the database does not include detailed clinical information such as symptoms, vital signs, urine output, laboratory data, cause and type of burn injury (e.g. flame, electrical, scald, etc.), and severity of inhalation injury. Although we were able to obtain data on total infusion volume, we were not able to calculate in-out fluid balance or gains/losses in overall body weight. However, previous studies showed that higher mortality in burn patients was associated with TBSA (or burn index), older age, tracheal intubation, and inhalation injury [11], which were included in our study. In addition, key factors in the Acute Physiologic and Chronic Health Evaluation (APACHE) scoring system [27], which is widely used as a predictor of mortality or severity adjustment, were included in our study (vasopressor use instead of mean arterial pressure, mechanical ventilation use instead of oxygenation, RRT instead of renal function, and Japan Coma Scale instead of mental status). With regard to burn patients, the APACHE score has not been thoroughly validated and therefore a validated predictor of severity of disease for burn patients admitted to intensive care units; the Simplified Acute Physiology Score (SAPS) II score may be utilized where possible [28]. Unfortunately, the SAPS II score was not retrievable from the database. Second, we had no standardized protocol for high-dose vitamin C administration and consequently a larger number of severely ill patients may have inadvertently received vitamin C. Although we adjusted for disease severity using propensity score analysis, this methodology cannot adjust for unmeasured confounders. Third, we could not obtain data on the exact time when patients were injured. Although vitamin C was administered at day 1 of admission in this study, we were ultimately unaware whether patients had been transferred to the hospital immediately after sustaining their injuries. Although we included transportation from another hospital as a variable in our propensity model, delayed discovery of patients or transportation to another hospital may have resulted in delayed administration of vitamin C. Fourth, we were unable to adjust for differences between hospitals. The hospital volume (number of burn patients) or proportions of patients who received high-dose vitamin C may have affected the outcomes. Fifth, in comparison to international burn cohorts, the cohort in this study is significantly older. One possible explanation lies in the fact that Japan is one of the most progressively aged societies in the world and therefore the relatively skewed population in favor of the elderly could have contributed to this outcome that differs from the international community. Another possible contributing factor to the trend of elderly patients sustaining greater burns stems from Japan’s cultural embrace of bathing in comparatively hotter temperatures—this is particularly true for the elderly population. The scalding bathwater could have impacted this interesting demographic. In a study conducted in Japan, bathtub-related burns displayed a tendency of affecting the elderly and in an extensive and deeper manner [29]. Sixth, the database utilized in the present study fails to include TBSA. As TBSA was not retrievable, the burn index that reflects both the surface area and the thickness of the burn was adopted. As described in the methodology, previous studies have confirmed that the burn index was a good predictor of mortality in burn patients [11, 15]. Finally, the database lacked data on long-term outcomes after discharge. We may not adjust the differences between hospitals. Nevertheless, the results of this study will provide the basis for larger and more well-designed randomized controlled trials.

## Conclusions

High-dose vitamin C therapy was associated with reduced in-hospital mortality in patients with severe burns under a minimum threshold of 10 g within the first 2 days of admission. While “high-dose” vitamin C therapy lacked a universal dosage definition, the present study endeavored to determine whether varying thresholds of “high-dose” vitamin C therapy can confer different degrees of survival advantages to severe burn patients. Although the results presented in this observational study offer unique insight into the controversy surrounding vitamin C administration in burn victims, it is imperative that additional prospective studies be conducted to provide further clarification on this debate.

## Supplementary information

**Additional files 1: Figure S1.** Patient selection (24 g minimum threshold of high-dose vitamin C).

**Additional files 2: Table S1.** Baseline patient characteristics before and after propensity score matching (24 g minimum threshold of high-dose vitamin C).

## Data Availability

Not applicable

## Abbreviations

ICD-10: International Classification of Diseases, Tenth Revision
JCS: Japan Coma Scale
CCI: Charlson comorbidity index
RRT: Renal replacement therapy
IQR: Interquartile range
APACHE: Acute Physiologic and Chronic Health Evaluation
