# Supplementary figures and images for: Effect of high-dose vitamin C therapy on severe burn patients: a nationwide cohort study
Source: Crit Care. 2019 Dec 12;23:407. doi: 10.1186/s13054-019-2693-1 (PMC6909452; doi:10.1186/s13054-019-2693-1)

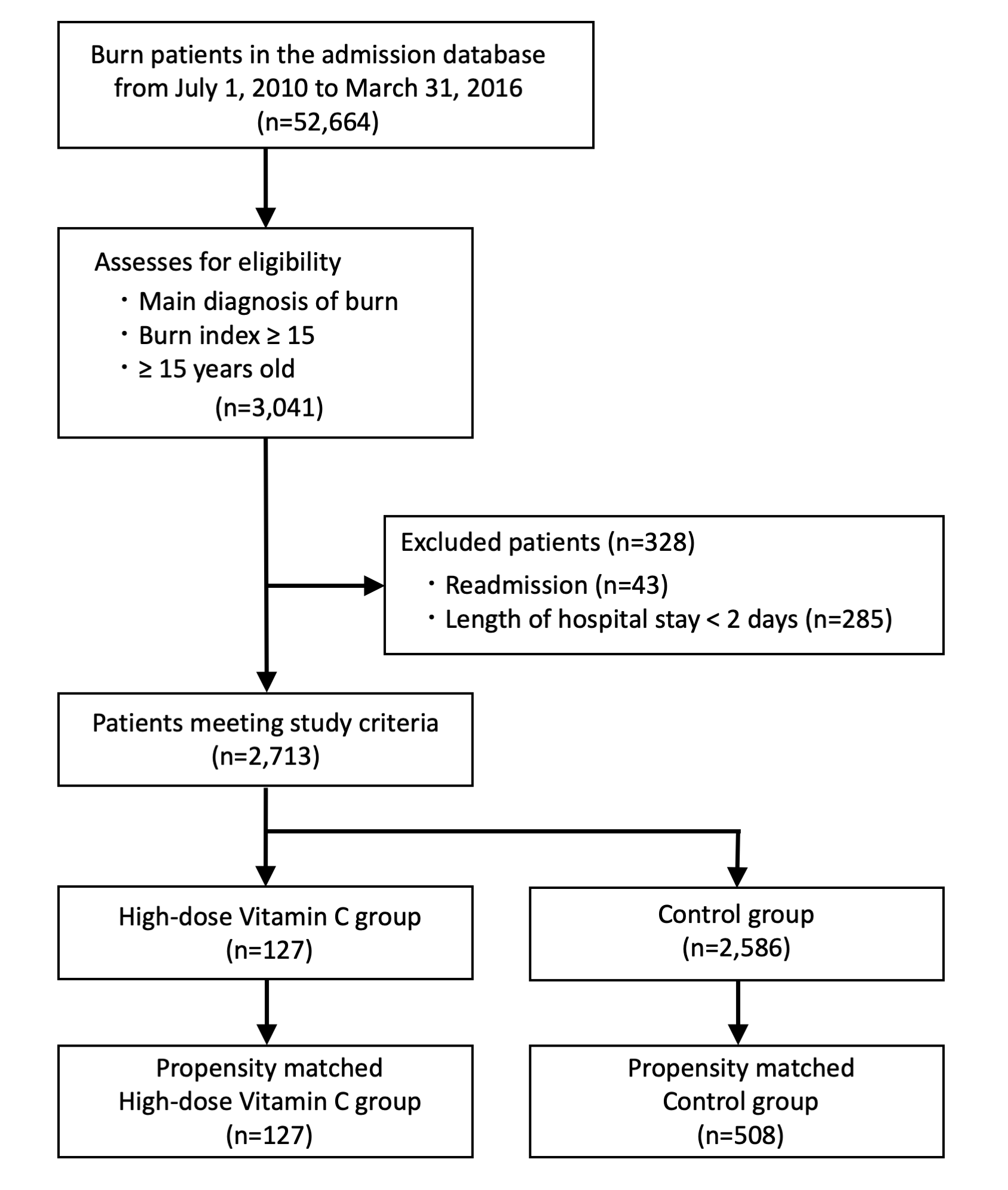

Supplement: Supplementary file 1 — Additional files 1: Figure S1. Patient selection (24 g minimum threshold of high-dose vitamin C). [file 13054_2019_2693_MOESM1_ESM.tiff]
