# Supplementary material for: Effect of high-dose vitamin C therapy on severe burn patients: a nationwide cohort study
Source: Crit Care. 2019 Dec 12;23:407. doi: 10.1186/s13054-019-2693-1 (PMC6909452; doi:10.1186/s13054-019-2693-1)
Supplement: Supplementary file 2 — Additional files 2: Table S1. Baseline patient characteristics before and after propensity score matching (24 g minimum threshold of high-dose vitamin C). [file 13054_2019_2693_MOESM2_ESM.doc]

**Table S1.** Baseline patient characteristics before and after propensity score matching (24 g minimum threshold of high-dose vitamin C)

|  | Before propensity score matching | | | | |  | After propensity score matching | | | | | |
| --- | --- | --- | --- | --- | --- | --- | --- | --- | --- | --- | --- | --- |
| Variables | High-dose  vitamin C group (n=157) | | Control group (n=2556) | | Standardized difference (%) |  | | High-dose  vitamin C group (n=157) | | Control group (n=628) | | Standardized difference (%) |
| Age(years), median (IQR) | 67 | (48-80) | 69 | (51-80) | 11.7 |  | | 67 | (48-80) | 67.5 | (50-80) | 7.6 |
| Male, n(%) | 87 | (55.4) | 1561 | (61.1) | 11.5 |  | | 87 | (55.4) | 332 | (52.9) | 5.1 |
| Body weight, median (IQR) | 58.5 | (47.4-68.9) | 55.4 | (45-65) | 23.4 |  | | 58.5 | (47.4-68.9) | 57.15 | (50-69) | 2.6 |
| Burn index, median (IQR) | 34 | (24-50) | 25 | (18-40) | 39.3 |  | | 34 | (24-50) | 34.5 | (23-53.4) | 1.1 |
| Inhalation injury, n(%) | 45 | (28.7) | 523 | (20.5) | 19.1 |  | | 45 | (28.7) | 181 | (28.8) | 0.4 |
| Charlson Cormobidity Index, n(%) |  |  |  |  |  |  | |  |  |  |  |  |
| 0 (low) | 126 | (80.3) | 1917 | (75.0) | 12.6 |  | | 126 | (80.3) | 484 | (77.1) | 7.8 |
| 1 (medium) | 24 | (15.3) | 444 | (17.4) | 5.6 |  | | 24 | (15.3) | 115 | (18.3) | 8.1 |
| ≥2 (high) | 7 | (4.5) | 195 | (7.6) | 13.3 |  | | 7 | (4.5) | 29 | (4.6) | 0.8 |
| Japan Coma Scale, n(%) |  |  |  |  |  |  | |  |  |  |  |  |
| 0 (alert) | 69 | (43.9) | 1406 | (55.0) | 22.3 |  | | 69 | (43.9) | 266 | (42.4) | 3.2 |
| 1-30 (dizziness) | 38 | (24.2) | 553 | (21.6) | 6.1 |  | | 38 | (24.2) | 145 | (23.1) | 2.6 |
| 10-30 (somnolence) | 6 | (3.8) | 173 | (6.8) | 13.2 |  | | 6 | (3.8) | 28 | (4.5) | 3.2 |
| 100-300 (coma) | 44 | (28.0) | 424 | (16.6) | 27.7 |  | | 44 | (28.0) | 189 | (30.1) | 4.6 |
| Prescriptions within 1 day, n(%) |  |  |  |  |  |  | |  |  |  |  |  |
| Vasopressor use | 44 | (28.0) | 441 | (17.3) | 25.9 |  | | 44 | (28.0) | 180 | (28.7) | 1.4 |
| Albumin use | 74 | (47.1) | 530 | (20.7) | 57.9 |  | | 74 | (47.1) | 285 | (45.4) | 3.5 |
| Hydroxyethyl starch use | 13 | (8.3) | 122 | (4.8) | 14.2 |  | | 13 | (8.3) | 43 | (6.8) | 5.4 |
| Intravenous antibiotics use | 70 | (44.6) | 1159 | (45.3) | 1.5 |  | | 70 | (44.6) | 289 | (46.0) | 2.9 |
| Neuromuscular blockade use | 63 | (40.1) | 565 | (22.1) | 39.6 |  | | 63 | (40.1) | 254 | (40.4) | 0.6 |
| Haptoglobin use | 59 | (37.6) | 378 | (14.8) | 53.6 |  | | 59 | (37.6) | 242 | (38.5) | 2.0 |
| Transfusion use | 34 | (21.7) | 212 | (8.3) | 38.0 |  | | 34 | (21.7) | 132 | (21.0) | 1.6 |
| Procedures within 1 day, n(%) |  |  |  |  |  |  | |  |  |  |  |  |
| Operation | 61 | (38.9) | 438 | (17.1) | 49.7 |  | | 61 | (38.9) | 243 | (38.7) | 0.3 |
| Mechanical ventilation | 114 | (72.6) | 1180 | (46.2) | 55.8 |  | | 114 | (72.6) | 474 | (75.5) | 6.5 |
| Renal replacement therapy | 8 | (5.1) | 71 | (2.8) | 11.9 |  | | 8 | (5.1) | 27 | (4.3) | 3.8 |
| Endoscopy | 54 | (34.4) | 448 | (17.5) | 39.1 |  | | 54 | (34.4) | 212 | (33.8) | 1.3 |
| Enteral feeding | 11 | (7.0) | 202 | (7.9) | 3.4 |  | | 11 | (7.0) | 47 | (7.5) | 1.8 |
| Intra-arterial blood pressure monitoring | 123 | (78.3) | 1161 | (45.4) | 71.9 |  | | 123 | (78.3) | 492 | (78.3) | 0.1 |
| Admission site, n(%) |  |  |  |  |  |  | |  |  |  |  |  |
| Teaching hospital | 156 | (99.4) | 2436 | (95.3) | 25.4 |  | | 156 | (99.4) | 623 | (99.2) | 1.9 |
| Intensive care unit | 155 | (98.7) | 2059 | (80.6) | 62.4 |  | | 155 | (98.7) | 622 | (99.0) | 3.0 |
| Transportation from another hospital, n(%) | 7 | (4.5) | 124 | (4.9) | 1.9 |  | | 7 | (4.5) | 35 | (5.6) | 5.1 |

IQR: interquartile range
